# Supplementary material for: Phospholipids are imported into mitochondria by VDAC, a dimeric beta barrel scramblase
Source: Nat Commun. 2023 Dec 8;14:8115. doi: 10.1038/s41467-023-43570-y (PMC10709637; doi:10.1038/s41467-023-43570-y)
Supplement: Supplementary file 1 — Supplementary Information [file 41467_2023_43570_MOESM1_ESM.pdf]

Phospholipids are imported into mitochondria by VDAC, a dimeric beta barrel scramblase

Helene Jahn[1], Ladislav Bartoš[2,3], Grace I. Dearden[1], Jeremy S. Dittman[1], Joost C. M. Holthuis[4], Robert Vácha[2,3,*], Anant K. Menon[1,*]

[1]Department of Biochemistry, Weill Cornell Medical College; New York, NY 10065, USA
[2]CEITEC - Central European Institute of Technology, Masaryk University; Kamenice 5, 62500 Brno, Czech Republic
[3]National Centre for Biomolecular Research, Faculty of Science, Masaryk University; Kamenice 5, 62500 Brno, Czech Republic
[4]Department of Molecular Cell Biology, University of Osnabrück; Osnabrück 49076, Germany
*Corresponding authors: E-mails: robert.vacha@muni.cz, akm2003@med.cornell.edu

SUPPLEMENTARY INFORMATION

SUPPLEMENTARY FIGURES S1-S13
MOVIE S1
SUPPLEMENTARY TABLES 1-8

SUPPLEMENTARY FIGURES S1-S13

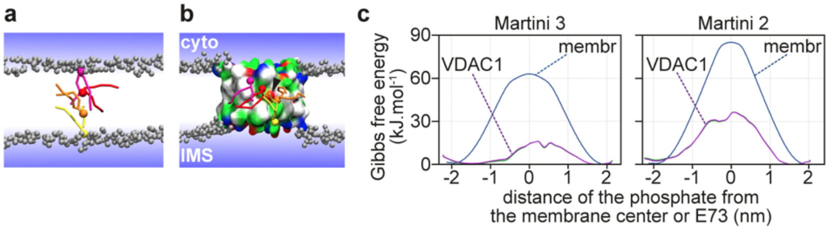

Figure S1. MD simulations to assess the energetics of phospholipid translocation in the presence of human VDAC1.

a, b. Transbilayer phospholipid translocation was analyzed by coarse-grained molecular dynamics simulations of a pure POPC bilayer and a POPC bilayer containing human VDAC1 (PDB ID 6G6U). A phospholipid was pulled from one leaflet of the bilayer to the other in the z-direction. In the case of VDAC1, the lipid phosphate was also restrained to be within a cylinder, centered on E73, with a radius of 1.5 nm. Five selected positions along the translocation pathway from the upper (IMS) to lower (cytoplasmic) side are shown colored from yellow to red (the lipid headgroup is represented by single bead, and acyl chains are represented as thin tubes). Membrane phospholipids are not shown explicitly - their phosphate groups are shown by grey beads, while the lipid tails are not displayed for clarity. VDAC1 is represented as a protein surface color-coded as follows: hydrophobic-white, polar-green, red-negatively charged, and blue-positively charged residues. **c.** Calculated free energy profiles of lipid translocation as a function of the distance between the transiting lipid phosphate and the membrane center or E73 are shown. The presence of VDAC1 leads to a significant decrease in the energy barrier for phospholipid translocation around E73 using coarse-grained Martini 3 (left) or Martini 2 (right) force-fields. The error is estimated < 2 $kJ.mol^{-1}$ for the pure POPC bilayer and < 5 $kJ.mol^{-1}$ for the system with VDAC based on the profile asymmetry.

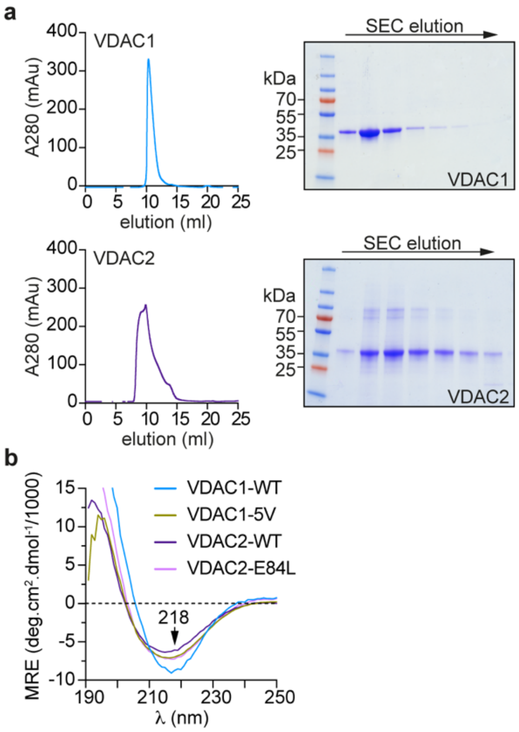

Figure S2. Purification of human VDAC1 and VDAC2.

a. Human VDAC proteins were produced heterologously in *E. coli*, purified as inclusion bodies, denatured with guanidine hydrochloride, solubilized using the detergent *N*,*N*-Dimethyldodecylamine *N*-oxide (LDAO) and purified by cation-exchange and size-exclusion chromatography (SEC). SEC profiles and associated Coomassie-stained SDS-PAGE analyses are shown. Additional SEC profles for VDAC1 are shown in Fig. S7a. The data in the paper are based on 5 independent VDAC1 preparations and 6 independent VDAC2 preparations. **b.** Circular Dichroism spectroscopy of human VDAC proteins. Spectra were obtained with 66 µM VDAC samples (wild-type (WT) VDAC1 and VDAC2, VDAC1-5V mutant, and VDAC2-E84L mutant) in a 0.2 mm sandwich cell. All proteins showed high beta-sheet content. Spectra of VDAC1 and VDAC2 were taken twice, other spectra were taken once.

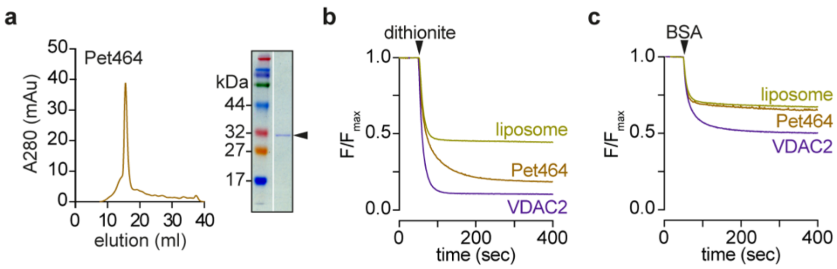

Figure S3. The Pet464 β-barrel protein does not scramble phospholipids.
a. Size-exclusion chromatography (SEC) profile of Pet464, the β-barrel portion of the Pet autotransporter. The β-barrel was produced in *E. coli*, recovered as inclusion bodies, denatured with urea and re-folded in LDAO detergent. The refolded protein was purified using Ni-NTA affinity chromatography and SEC. The SEC profile and Coomassie-stained SDS-PAGE analysis of the peak fraction are shown; the arrowhead indicates the ~30 kDa purified protein. Pet464 was purified on two separate occasions; a third sample of Pet464 was a gift of Matthew Johnson and Denisse Leyton (Australian National University). **b, c.** Dithionite (panel b) and BSA-back extraction (panel c) assay traces comparing Pet464 with VDAC2 in vesicles containing NBD-PC as the reporter lipid. Both proteins were reconstituted at a predicted density of 60 copies per vesicle. Normalized fluorescence traces are shown. These assays were performed 4 times.

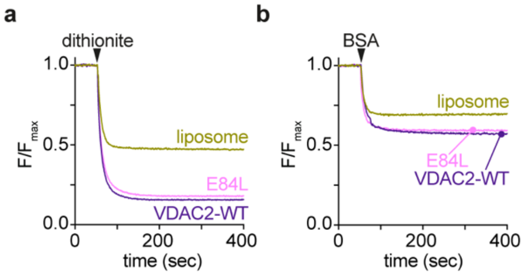

Figure S4. VDAC2 E84L is a scramblase.
VDAC2-E84L was purified as in Figure S2 (CD spectrum shown in Fig. S2b), reconstituted into unilamellar vesicles containing NBD-PC at a theoretical protein/vesicle copy number of 10, and assayed for channel and scramblase activity. Wild-type VDAC2 was analyzed in parallel. **a.** Channel assay, as described in Fig. 1d. **b.** Scramblase assay, as described in Fig. 1f. These assays were performed 3 times.

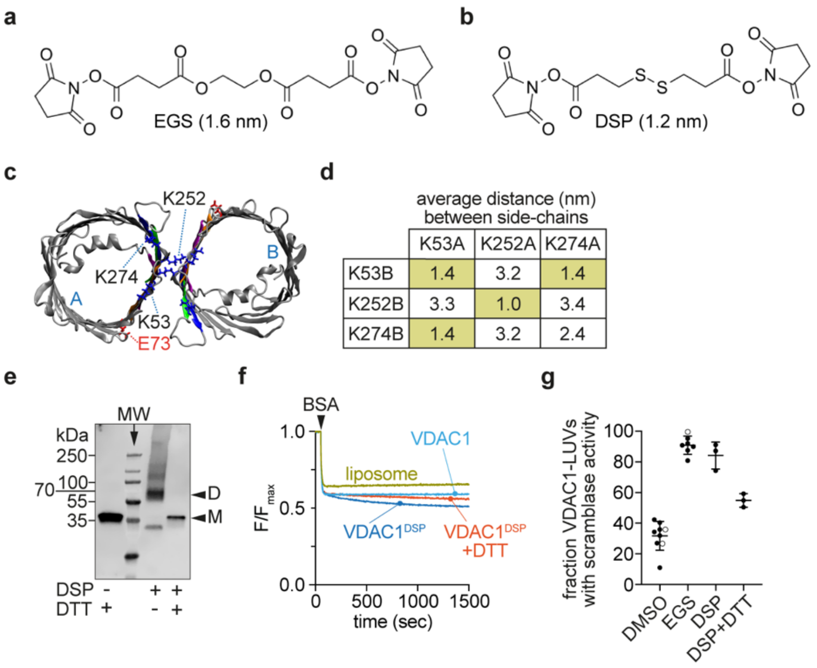

Figure S5. Scramblase activity of VDAC1 dimers prepared with a cleavable crosslinker.
a. Chemical structure of EGS. **b.** Chemical structure of DSP. **c.** Top view of dimer-1, indicating outward-facing lysine residues in protomers designated A and B. **d.** Distances between lysine pairs on VDAC1 protomers in dimer-1 calculated from a coarse-grained MD simulation that accounts for reorientation of the sidechains. Each of the indicated distances is the average xyz distance between the sidechain bead of lysines on protomers A and B. The shaded boxes indicate lysine pairs that are <1.4 nm apart and therefore likely to be crosslinked by EGS and DSP. **e.** VDAC1 in LDAO was cross-linked using DSP or mocked treated with DMSO (indicated (-)) and visualized by SDS-PAGE immunoblotting using antibodies against the N-terminal His tag. An aliquot of the DSP-cross-linked sample was treated with dithiothreitol (DTT) after reconstitution into liposomes and analyzed alongside. This experiment was done 3 times with similar results. **f.** Cross-linked (VDAC^{DSP}) or mock-treated (VDAC1) proteins were reconstituted into LUVs at a copy number of 30 proteins/vesicle and assayed for scramblase activity using BSA back extraction as in Fig. 1f. Normalized fluorescence traces are shown. Also shown is an assay in which a VDAC^{DSP}-reconstituted sample was treated with DTT to reverse the crosslink *in situ*. **g.** Fraction of VDAC1-vesicles with scramblase activity; data shown correspond to reconstituted DMSO-treated VDAC1, reconstituted EGS- or DSP-crosslinked VDAC1, and VDAC1^{DSP} treated with DTT after reconstitution at a theoretical protein/vesicle copy number of 30. Open circles in the DMSO and EGS data represent samples that were treated with DTT as a control. Data represent mean ± SD, based on at least three independent reconstitutions.

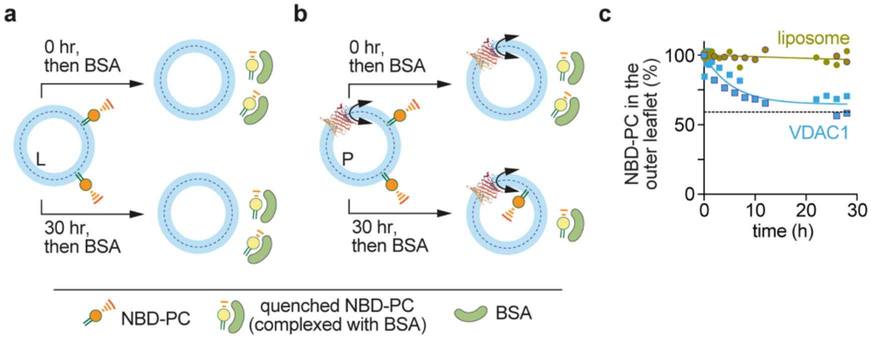

Figure S6. VDAC1 monomers scramble phospholipids slowly.

a, b. Schematic of assay to detect slow scrambling. Asymmetric liposomes were prepared by adding 0.25 mol% NBD-PC lipids in EtOH (final EtOH concentration 0.4%) to protein-free liposomes (L) (panel a) or VDAC1-containing proteoliposomes (P) reconstituted at a theoretical protein/vesicle copy number of 10 (panel b). Samples were incubated for 30 min on ice after which they were incubated for approximately 30 h at 20°C. Aliquots were taken at different time points to perform a BSA-back extraction assay and determine the fraction of NBD-lipids present in the outer leaflet. If a slow scramblase is present, over the time course of 30 h, approximately 50% of NBD lipids should be shielded from BSA extraction at the inner leaflet. **c.** Slow scrambling by VDAC1. Two independent experiments with VDAC1-proteoliposomes are shown, fitted to a single exponential decay function. Parallel experiments with protein-free liposomes are also shown. The black dotted line indicates the expected fluorescence signal for the VDAC1-proteoliposomes, experimentally determined from protein reconstitutions with same conditions. The entire complement of NBD-PC molecules in the protein-free vesicles was accessible for BSA-extraction over the time-course of the experiment, indicating little or no scrambling. In contrast, for vesicles reconstituted with hVDAC1, nearly 50% of the NBD-lipids were shielded from BSA-extraction after 20 h, indicating a scrambling half-life ~4 h.

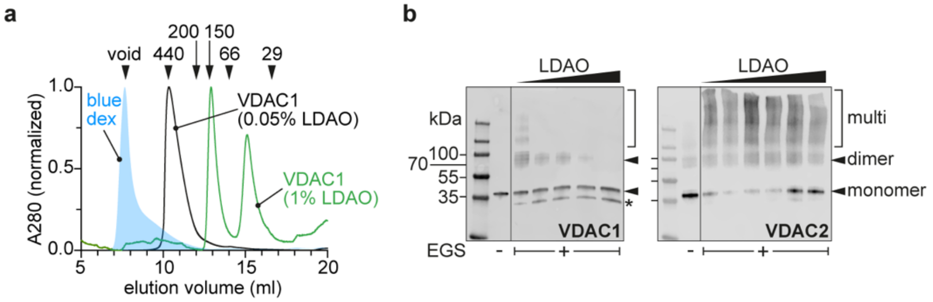

Figure S7. SEC analysis and crosslinking of LDAO-solubilized VDAC proteins.

a. Size exclusion chromatography of VDAC1 in 0.05% or 1% LDAO. The chromatogram generated in 0.05% LDAO is also shown in Fig. S2. For the sample analyzed in 1% LDAO, VDAC1 prepared in 0.05% LDAO was diluted into buffer with 1% LDAO and incubated for 45 min at 37°C prior to analysis. Migration of molecular weight standards is indicated (in units of kDa); the profile of blue dextran (blue dex) marks the void volume. **b.** The oligomeric state of VDAC1 (left panel) and VDAC2 (right panel) in different concentrations of LDAO (0.05% - 1.05% w/v) was determined by cross-linking using the homobifunctional, amine-reactive crosslinker EGS at a 16-fold molar excess over lysine residues in the protein. EGS was delivered from a stock solution in DMSO (final DMSO concentration = 0.5%) and the reaction was carried out for 40 min at 20°C. Control samples (indicated (-)) were mock-treated with DMSO. The crosslinking reactions were analyzed by SDS-PAGE immunoblotting with anti-His tag antibodies. Oligomerization is highly dependent on the detergent concentration with mainly (VDAC1) or a greater proportion (VDAC2) of monomers present at high detergent concentrations. This experiment was done 2 times.

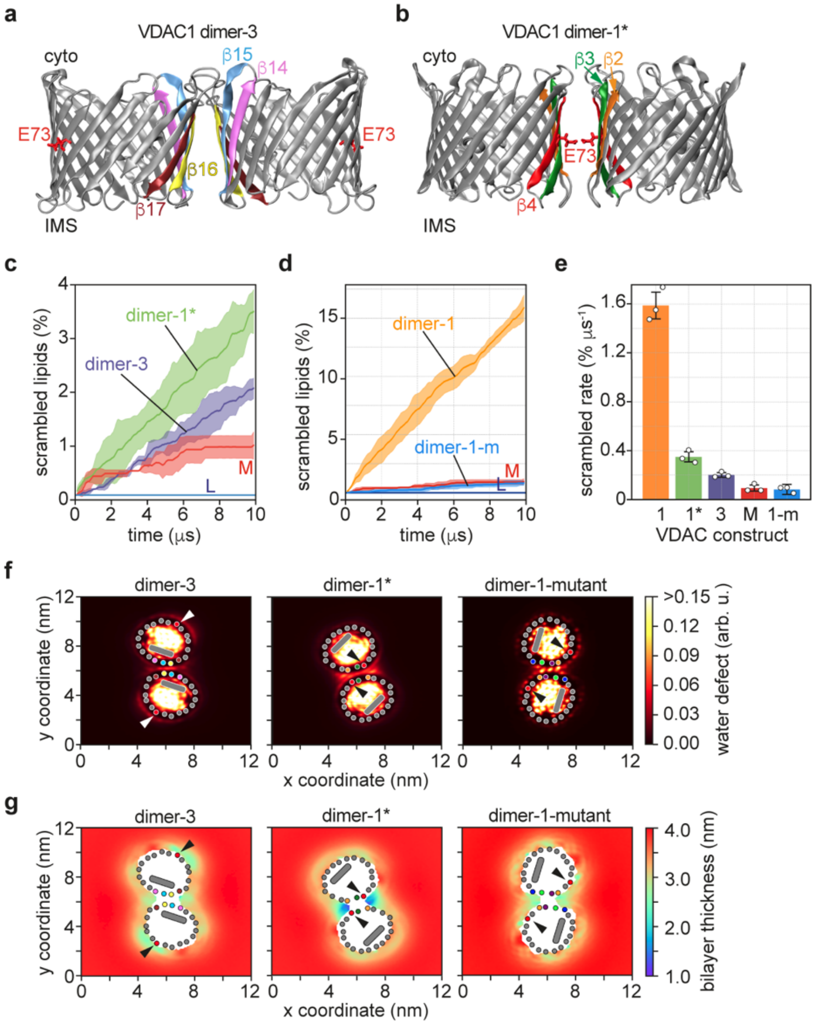

Figure S8. Structures of various VDAC1 dimers and their effects on the membrane.
a. Structure of VDAC1 dimer-3. The β-strands at the interface are colored as indicated, and the E73 residue on the β4-strand is marked. **b.** Structure of VDAC1 dimer-1*; details are as in panel a. **c.** Percentage of lipids scrambled as a function of simulation time. The graphs show the scrambling activity of dimer-1*, dimer-3, monomer (M), and protein-free membrane (L) (running average over 200 ns time intervals, shading=68% confidence interval, n=3). **d.** As in panel c for constrained dimer-1 (taken from Fig. 4c) and constrained dimer-1-mutant (dimer-1-m). The mutations in dimer-1-m are T77V, S43V, T33V, S35V, Y247V, Q249V. **e.** Bar chart showing the scrambling rate for dimer-1, -1*, -3, monomer (M) (taken from Fig. 4d), and dimer-1-m (labeled 1-m)(mean ± SD, n=3). **f.** Top views of water penetration into the membrane (water defect, color LUT shown at right) in the vicinity of the dimer interfaces. The β-strands at the interface are indicated as colored dots (same color scheme as in panels a, b); the E73 residue is shown as a red dot and marked by arrowheads; the N-terminal helix is shown as a grey oblong within the VDAC1 pore. **g.** Bilayer thinning at the dimer interfaces (thickness scale, color LUT shown at right, E73 indicated by arrowhead). Other details are as in panel f.

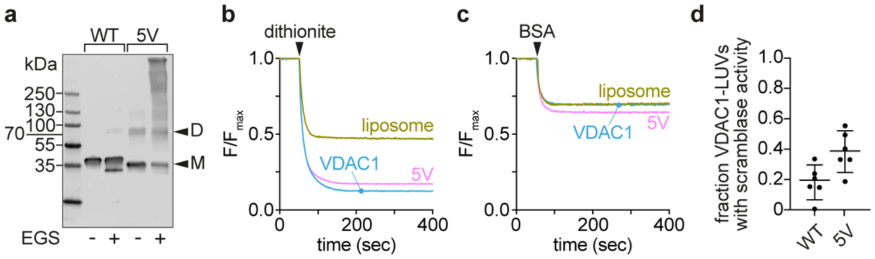

Figure S9. Analysis of the VDAC1-5V mutant.

a. VDAC1-5V is a dimer/oligomer in liposomes. VDAC1 WT and the 5V mutant were reconstituted into unilamellar vesicles at a theoretical protein/vesicle copy number of 30 and subjected to EGS crosslinking to determine their oligomeric state. Control samples were mocked treated with DMSO. The samples were analyzed by SDS-PAGE immunoblotting using antibodies against the N-terminal His tag, revealing the 5V mutant to be highly dimeric/oligomeric. **b.** Representative channel assay as described in Fig. 1d of VDAC1-WT and VDAC1-5V reconstituted to have 10 proteins/vesicle on average. **c.** Representative scramblase assay as described in Fig. 1f of VDAC1 WT and VDAC1-5V of samples prepared in panel b. **d.** Fraction of VDAC1-vesicles with scramblase activity (calculated from data similar to and including those shown in panels b and c; n=6 independent reconstitutions). Data represent mean ± 95% CI. Despite its dimeric/multimeric nature after reconstitution as evidenced by EGS crosslinking, the 5V mutant is not an effective scramblase.

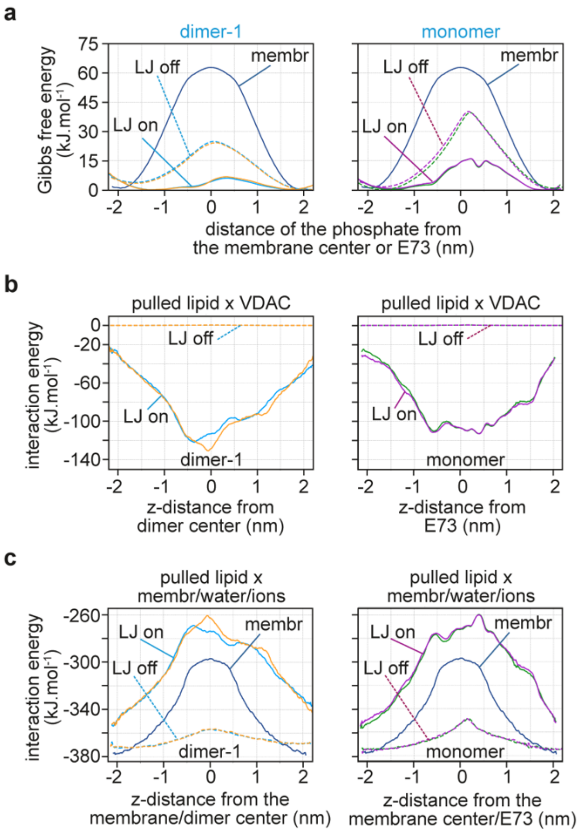

Figure S10. The effect of VDAC1 and membrane disruption on the energetics of lipid scrambling.

a. Free energy profiles of phospholipid translocation as a distance between the lipid phosphate and the membrane center in pure POPC membrane (blue line), in the presence of VDAC1 monomer (green and magenta lines in the right panel), and in the presence of dimer-1 (azure and orange lines in the left panel). Dashed lines correspond to simulations in which the attractive dispersion interactions (Lennard-Jones (LJ) interactions) between the lipid and VDAC1 were turned off, effectively removing the direct contribution of VDAC1 to the decrease in the flip-flop barrier. **b,c.** Average enthalpic interactions (Lennard-Jones + electrostatic) between the scrambling lipid and VDAC (b) or between the scrambling lipid and membrane + water + ions (c) as a distance between the scrambling lipid phosphate and the membrane center. The enthalpic interactions were calculated from the simulated umbrella sampling windows. The color scheme is the same as in panel a.

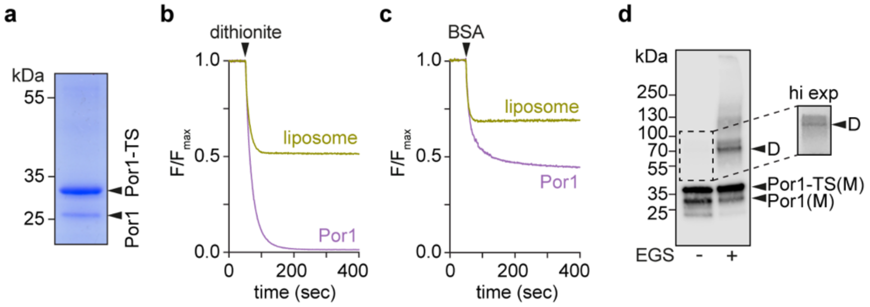

Figure S11. Native yeast VDAC (Por1) scrambles phospholipids.
a. Coomassie-stained SDS-PAGE of Twin-Strep-tagged yeast VDAC (Por1-TS). The construct was expressed in W303 yeast cells, extracted from cell membranes in LDAO detergent, and purified on Strep-Tactin XT resin, followed by size-exclusion chromatography. Mass spectrometry confirmed that both bands on the gel correspond to Por1: the strongly stained upper band is Por1-TS, and the lower band is endogenous Por1, which copurifies with Por1-TS. The term 'native' here signifies that this protein was extracted from cells in folded form, in contrast to the human VDAC1 and VDAC2 preparations (Fig. S2) where protein was recovered in inclusion bodies, denatured and re-folded. **b, c.** Normalized fluorescence traces corresponding to dithionite (panel b) and BSA-back extraction (panel c) assays performed on mock-treated liposomes or vesicles reconstituted with 12 ug of Por1-TS. Data points are shown as the mean of technical replicates (n = 3). **d.** Crosslinking of Por1 after reconstitution into vesicles. Reconstituted LUVs were treated with EGS crosslinker. The samples were analyzed by SDS-PAGE immunoblotting using anti-Por1 antibodies. Reconstituted Por1 shows a significant population of dimers/multimers on crosslinking. In the absence of EGS, high-exposure (hi-exp) of the blot reveals the presence of SDS-resistant Por1 dimers. The data shown here represent a single preparation of Por1-TS, analyzed once.

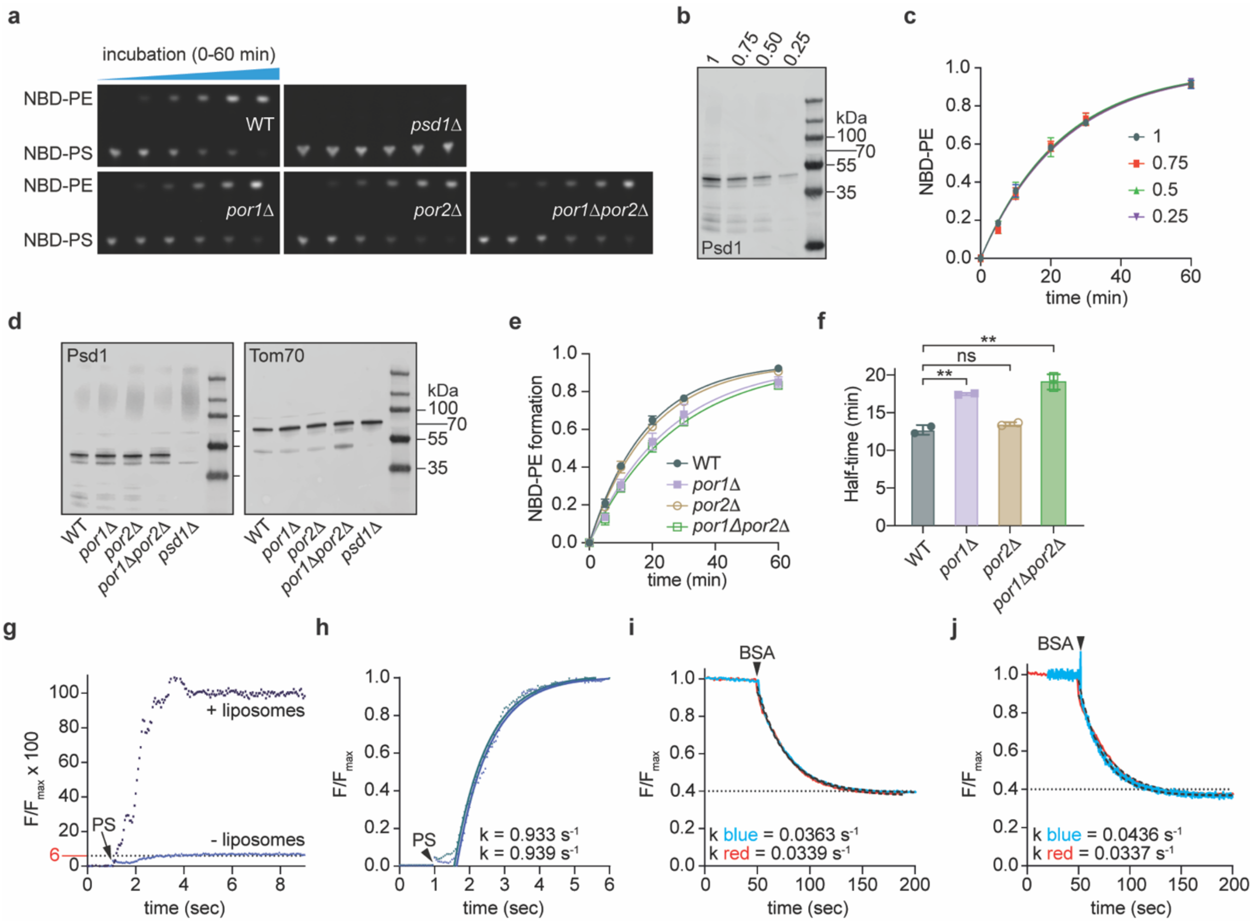

Figure S12. Supporting data for the PS-PE decarboxylation assay.
a. Thin layer chromatograms, visualized with a ChemiDoc imager, of decarboxylation assay time courses using mitochondria from the indicated yeast strains. These assays were done 5 times. **b.** Different concentrations of mitochondria from wild-type cells (0.25-1 mg/ml, as indicated) were prepared for the decarboxylation assay. Equivalents were analyzed by immunoblotting using anti-Psd1 antibodies. **c.** Time-course of NBD-PS decarboxylation at 30°C using mitochondria at different concentrations as shown in panel c supplemented with 1 µM NBD-PS at time=0 min. The data points are shown as mean ± SD, fitted to a single exponential function. The rate of NBD-PE production was not affected by lowering the amount of mitochondrial protein 4-fold, confirming that mitochondria are not limiting in the assay. **d.** Mitochondria were isolated from the indicated yeast strains, and adjusted to have the same protein concentration, confirmed by immunoblotting using antibodies against the OMM protein Tom70 and the IMM protein Psd1. Immunoblotting was performed at least once for each biological replica. **e.** Time-course of NBD-PS decarboxylation at 30°C using mitochondria (1 mg/mL) from the indicated yeast strains, supplemented with 1 µM NBD-PS at time=0 min. The data points - obtained from chromatograms such as those shown in panel a - are given as mean ± SD and fitted to a single exponential function (2 biological replicates, with at least 2 technical replicates per assay). No NBD-PE was detected in *psd1Δ* cells (panel a). **f.** Half-time of NBD-PS to NBD-PE conversion obtained by mono-exponential fitting of the time traces shown in panel e (mean ± SD, p ≈ 0.0021

(One-Way ANOVA followed up with multiple comparison test, corrected using Bonferroni)). Fitting and analysis was carried out using GraphPad Prism. **g.** Fluorescence difference between NBD-PS monomer and NBD-PS in a POPC:POPG membrane, determined by adding 0.1 µM NBD-PS to liposomes (+liposomes) or buffer (-liposomes). After background subtraction, the traces were normalized to the maximum fluorescence (F_{max}) of the +liposome sample. This measurement was done once. **h.** k_0 was determined by adding 1 µM NBD-PS to POPC:POPG liposomes and fitting the fluorescence increase to a single exponential function. Two individual traces, and associated rate constants (k) are shown. These measurements were performed 4 times. **i.** BSA back extraction of asymmetrically labeled liposomes (1 µM NBD-PS total in sample) was performed to determine k_1. Two traces are shown with different BSA concentrations (40 µl of 75 mg/ml BSA in red, or 40 µl of 37.5 mg/ml BSA in blue). The traces were analyzed using one-phase exponential decay; rate constants (k) are shown. Each trace was obtained twice. **j.** BSA back extraction of asymmetrically labeled liposomes as in panel i, except that liposomes were prepared with either 1 µM NBD-PS (red trace) or 0.05 µM NBD-PS (blue trace), the latter to prevent micelle formation. Traces were analyzed as in panel i, and the calculated rate constants are shown. Trace representative on n=5.

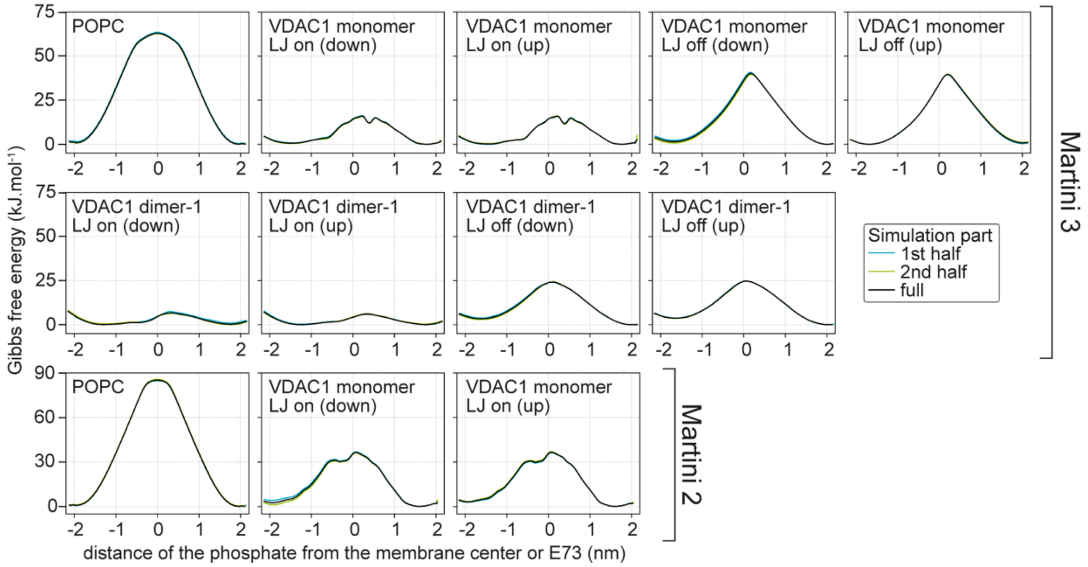

Figure S13. Supporting data for Figures S1 and S10 - convergence of free energy calculations. Convergence of free energy calculations captured by calculating additional free energy profiles from the first (azure) and the second (lime) halves of the production part of the umbrella sampling. The black profiles correspond to free energy profiles shown in Figs. S1 and S10.

MOVIE S1

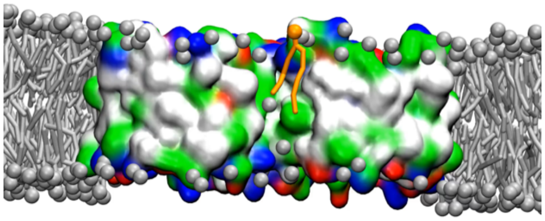

Movie S1. Phospholipid scrambling at the dimer-1 interface.
Movie capturing spontaneous scrambling of a lipid along the interface of dimer-1 in a coarse-grained molecular dynamics simulation. The dimer is shown using molecular surface, the headgroups of most lipids are shown as gray beads and their tails as gray tubes, while a single lipid is highlighted in orange. Most lipids in front of the dimer are not depicted, except for several phosphate groups that are located close to the dimer. The movie captures roughly 140 ns of simulated time. The image is a screen grab of the first frame of the movie.

SUPPLEMENTARY TABLES 1-8

Table 1. Distribution of umbrella sampling windows along the collective variable with the biasing force constants used for Martini 3 and Martini 2 free energy calculations of lipid scrambling in a protein-free membrane.

Window No.	Reference position [nm]	Force constant [kJ mol^{-1} nm^{-2}]	Window No.	Reference position [nm]	Force constant [kJ mol^{-1} nm^{-2}]
1	2.00	3000	25	−0.05	5000
2	1.90	3000	26	−0.10	5000
3	1.80	3000	27	−0.15	5000
4	1.70	3000	28	−0.20	5000
5	1.60	3000	29	−0.25	5000
6	1.50	3000	30	−0.30	3000
7	1.40	3000	31	−0.40	3000
8	1.30	3000	32	−0.50	3000
9	1.20	3000	33	−0.60	3000
10	1.10	3000	34	−0.70	3000
11	1.00	3000	35	−0.80	3000
12	0.90	3000	36	−0.90	3000
13	0.80	3000	37	−1.00	3000
14	0.70	3000	38	−1.10	3000
15	0.60	3000	39	−1.20	3000
16	0.50	3000	40	−1.30	3000
17	0.40	3000	41	−1.40	3000
18	0.30	3000	42	−1.50	3000
19	0.25	5000	43	−1.60	3000
20	0.20	5000	44	−1.70	3000
21	0.15	5000	45	−1.80	3000
22	0.10	5000	46	−1.90	3000
23	0.05	5000	47	−2.00	3000
24	0.00	5000			

Table 2. Distribution of umbrella sampling windows along the collective variable with the biasing force constants used for Martini 3 free energy calculations of lipid scrambling along monomeric VDAC1.

Window No.	Reference position [nm]	Force constant [kJ mol^{-1} nm^{-2}]	Window No.	Reference position [nm]	Force constant [kJ mol^{-1} nm^{-2}]
1	1.90	1000	33	0.00	4000
2	1.80	1000	34	−0.04	4000
3	1.70	1000	35	−0.08	4000
4	1.60	1000	36	−0.12	4000
5	1.50	1000	37	−0.16	4000
6	1.40	1000	38	−0.20	4000
7	1.30	1000	39	−0.24	4000
8	1.20	1000	40	−0.28	4000
9	1.10	1000	41	−0.32	4000
10	1.00	1000	42	−0.36	4000
11	0.95	2000	43	−0.40	4000
12	0.90	2000	44	−0.40	4000
13	0.85	2000	45	−0.48	4000
14	0.80	2000	46	−0.52	4000
15	0.75	2000	47	−0.56	4000
16	0.70	2000	48	−0.60	2000
17	0.65	2000	49	−0.65	2000
18	0.60	2000	50	−0.70	2000
19	0.56	4000	51	−0.75	2000
20	0.52	4000	52	−0.80	2000
21	0.48	4000	53	−0.90	1000
22	0.44	4000	54	−1.00	1000
23	0.40	4000	55	−1.10	1000
24	0.36	4000	56	−1.20	1000
25	0.32	4000	57	−1.30	1000
26	0.28	4000	58	−1.40	1000
27	0.24	4000	59	−1.50	1000

28	0.20	4000	60	−1.60	1000
29	0.16	4000	61	−1.70	1000
30	0.12	4000	62	−1.80	1000
31	0.08	4000	63	−1.90	1000
32	0.04	4000	64	−2.00	1000

Table 3. Distribution of umbrella sampling windows along the collective variable with the biasing force constants used for Martini 2 free energy calculations of lipid scrambling along monomeric VDAC1.

Window No.	Reference position [nm]	Force constant [kJ mol^{-1} nm^{-2}]	Window No.	Reference position [nm]	Force constant [kJ mol^{-1} nm^{-2}]
1	1.90	3000	37	0.00	5000
2	1.80	3000	38	−0.04	5000
3	1.70	3000	39	−0.08	5000
4	1.60	3000	40	−0.12	5000
5	1.50	3000	41	−0.16	5000
6	1.40	3000	42	−0.20	5000
7	1.30	3000	43	−0.24	5000
8	1.20	3000	44	−0.28	5000
9	1.10	3000	45	−0.32	5000
10	1.00	3000	46	−0.36	5000
11	0.95	3000	47	−0.40	5000
12	0.90	3000	48	−0.44	5000
13	0.85	3000	49	−0.46	5000
14	0.80	5000	50	−0.48	7000
15	0.75	5000	51	−0.50	7000
16	0.73	7000	52	−0.52	7000
17	0.70	7000	53	−0.54	5000
18	0.68	7000	54	−0.58	5000
19	0.66	7000	55	−0.60	3000
20	0.64	7000	56	−0.65	3000
21	0.62	7000	57	−0.70	3000
22	0.60	5000	58	−0.75	3000
23	0.56	5000	59	−0.80	3000
24	0.52	5000	60	−0.90	3000
25	0.48	5000	61	−1.00	3000
26	0.44	5000	62	−1.10	3000
27	0.40	5000	63	−1.20	3000

28	0.36	5000	64	−1.30	3000
29	0.32	5000	65	−1.40	3000
30	0.28	5000	66	−1.50	3000
31	0.24	5000	67	−1.60	3000
32	0.20	5000	68	−1.70	3000
33	0.16	5000	69	−1.80	3000
34	0.12	5000	70	−1.90	3000
35	0.08	5000	71	−2.00	3000
36	0.04	5000	72	−2.10	3000

Table 4. Distribution of umbrella sampling windows along the collective variable with the biasing force constants used for Martini 3 free energy calculations of lipid scrambling along dimer-1.

Window No.	Reference position [nm]	Force constant [kJ mol^{-1} nm^{-2}]	Window No.	Reference position [nm]	Force constant [kJ mol^{-1} nm^{-2}]
1	1.80	50	9	−0.20	200
2	1.40	50	10	−0.40	200
3	1.00	50	11	−0.60	100
4	0.80	100	12	−0.80	100
5	0.60	100	13	−1.00	50
6	0.40	200	14	−1.40	50
7	0.20	200	15	−1.80	50
8	0.00	200			

Table 5. Distribution of umbrella sampling windows along the collective variable with the biasing force constants used for Martini 3 free energy calculations of lipid scrambling along VDAC1 monomer and dimer-1 with attractive LJ interactions turned off.

Window No.	Reference position [nm]	Force constant [kJ mol^{-1} nm^{-2}]	Window No.	Reference position [nm]	Force constant [kJ mol^{-1} nm^{-2}]
1	2.20	1000	33	0.04	4000
2	2.10	1000	34	0.00	4000
3	2.00	1000	35	−0.04	4000
4	1.90	1000	36	−0.08	4000
5	1.80	1000	37	−0.12	4000
6	1.70	1000	38	−0.16	4000
7	1.60	1000	39	−0.20	4000
8	1.50	1000	40	−0.24	4000
9	1.40	1000	41	−0.28	4000
10	1.30	1000	42	−0.32	4000
11	1.20	1000	43	−0.36	4000
12	1.10	1000	44	−0.40	4000
13	1.00	1000	45	−0.44	4000
14	0.90	1000	46	−0.48	4000
15	0.80	1000	47	−0.52	4000
16	0.75	2000	48	−0.56	4000
17	0.70	2000	49	−0.60	2000
18	0.65	2000	50	−0.65	2000
19	0.60	2000	51	−0.70	1000
20	0.56	4000	52	−0.80	1000
21	0.52	4000	53	−0.90	1000
22	0.48	4000	54	−1.00	1000
23	0.44	4000	55	−1.10	1000
24	0.40	4000	56	−1.20	1000
25	0.36	4000	57	−1.30	1000
26	0.32	4000	58	−1.40	1000
27	0.28	4000	59	−1.50	1000
28	0.24	4000	60	−1.60	1000
29	0.20	4000	61	−1.70	1000
30	0.16	4000	62	−1.80	1000
31	0.12	4000	63	−1.90	1000
32	0.08	4000	64	−2.00	1000

Table 6. Lipid composition, number of water beads, ion concentration, total number of beads, and approximate box dimensions for all simulated systems. Note that one Martini bead corresponds to 4 molecules of water.

system	lipid composition	number of water beads	KCl concentration [mol dm^{-3}]	total number of beads	box dimensions
POPC (Martini 3)	256 POPC	5137	0.1	8285	9 × 9 × 11
VDAC1 monomer (Martini 3)	654 POPC	15,674	0.1	24,404	15 × 15 × 12
VDAC1 dimer-1 (Martini 3)	918 POPC	19,601	0.1	32,207	18 × 18 ×11
VDAC1 dimer-1* (Martini 3)	920 POPC	19,809	0.1	32,443	18 × 18 ×11
VDAC1 dimer-3 (Martini 3)	916 POPC	19.785	0.1	32,371	18 × 18 ×11
VDAC1 dimer-1-m (Martini 3)	924 POPC	18,960	0.1	31,624	18 × 18 ×11
POPC (Martini 2)	256 POPC	5212	0.1	8414	9 × 9 × 11
VDAC1 monomer (Martini 2)	640 POPC	15,101	0.1	23,751	15 × 15 × 12

Table 7. Yeast strains used in this study.

Strain name	Genetic Background	Mating Type	Genotype	Source
TKY705 (Wild Type)	W303	a	ura3-52 trp1Δ2 leu2-3_112 his3-11 ade2-1 CAN1	Miyata et al. (2018)
TKY705 psd1Δ	W303	a	TKY705, psd1Δ::natMX4	This study
TKY705 por1Δ	W303	a	TKY705, por1Δ::natMX4	This study
TKY705 por2Δ	W303	a	TKY705, por2Δ::natMX4	This study
TKY705 por1Δpor2Δ	W303	a	TKY705, por1Δ::natNT2 por2Δ::hphNT1	Miyata et al. (2018)

Miyata N, Fujii S, Kuge O (2018) Porin proteins have critical functions in mitochondrial phospholipid metabolism in yeast. J Biol Chem 293: 17593-17605

Table 8. PCR templates and primers used in this study.

Locus	Template plasmid	Primer (5′ to 3′)
psd1Δ::natMX4	p4339	1: AAT CCT TCT TGG TCG TTA TTT TTT GAA GAA GAA GGA AAA GCA AAG CCA GCG ACA TGG AGG CCC AGA ATA C<br>2: GAT AAT ACT ATA TAC AGC AAA ATA AAT GCT AAC TTT ACA TAT GAT TGC TTC AGT ATA GCG ACC AGC ATT C
por1Δ::natMX4	p4339	1: ACG AAA CAG CCA AGC GTA CCC AAA GCA AAA ATC AAA CCA ACC TCT CAA CAG ACA TGG AGG CCC AGA ATA C<br>2: GGA AAC AAG AAC GAG CAC ATA TAT GGT ATA TAG TGA ACA TAT ATA TAT TAG ATA TAT ACG TCA GTA TAG CGA CCA GCA TTC
por2Δ::natMX4	p4339	1: TCT CGA GTG AAG GAA TTG CAT GAA GAG GAA AGT GTT AGA AAT TAC CTA CGG ACA TGG AGG CCC AGA ATA C<br>2: TCA AAA GTA CAA ATC CTT ATT TAG TAA AAT TTA TAA GAA AAT AAA ATC TCC AGT ATA GCG ACC AGC ATT CA
