# Supplementary material for: Phospholipids are imported into mitochondria by VDAC, a dimeric beta barrel scramblase
Source: Nat Commun. 2023 Dec 8;14:8115. doi: 10.1038/s41467-023-43570-y (PMC10709637; doi:10.1038/s41467-023-43570-y)
Supplement: Supplementary file 3 — Description of Additional Supplementary Files [file 41467_2023_43570_MOESM3_ESM.pdf]

Description of Additional Supplementary Files

File Name: Supplementary Movie 1
Description: **Phospholipid scrambling at the dimer-1 interface.**
Movie capturing spontaneous scrambling of a lipid along the interface of dimer-1 in a coarsegrained molecular dynamics simulation. The dimer is shown using molecular surface, the headgroups of most lipids are shown as gray beads and their tails as gray tubes, while a single lipid is highlighted in orange. Most lipids in front of the dimer are not depicted, except for several phosphate groups that are located close to the dimer. The movie captures roughly 140 ns of simulated time. The image is a screen grab of the first frame of the movie.
