# Supplementary material for: Phospholipids are imported into mitochondria by VDAC, a dimeric beta barrel scramblase
Source: Nat Commun. 2023 Dec 8;14:8115. doi: 10.1038/s41467-023-43570-y (PMC10709637; doi:10.1038/s41467-023-43570-y)
Supplement: Supplementary file 5 — Reporting Summary [file 41467_2023_43570_MOESM5_ESM.pdf]

nature portfolio

Corresponding author(s): Anant K Menon

Last updated by author(s): Nov 9, 2023

Reporting Summary

Nature Portfolio wishes to improve the reproducibility of the work that we publish. This form provides structure for consistency and transparency in reporting. For further information on Nature Portfolio policies, see our Editorial Policies and the Editorial Policy Checklist.

Statistics

For all statistical analyses, confirm that the following items are present in the figure legend, table legend, main text, or Methods section.

n/a	Confirmed	
☐	☒	The exact sample size (*n*) for each experimental group/condition, given as a discrete number and unit of measurement
☐	☒	A statement on whether measurements were taken from distinct samples or whether the same sample was measured repeatedly
☐	☒	The statistical test(s) used AND whether they are one- or two-sided<br>*Only common tests should be described solely by name; describe more complex techniques in the Methods section.*
☒	☐	A description of all covariates tested
☒	☐	A description of any assumptions or corrections, such as tests of normality and adjustment for multiple comparisons
☐	☒	A full description of the statistical parameters including central tendency (e.g. means) or other basic estimates (e.g. regression coefficient) AND variation (e.g. standard deviation) or associated estimates of uncertainty (e.g. confidence intervals)
☐	☒	For null hypothesis testing, the test statistic (e.g. *F*, *t*, *r*) with confidence intervals, effect sizes, degrees of freedom and *P* value noted<br>*Give P values as exact values whenever suitable.*
☒	☐	For Bayesian analysis, information on the choice of priors and Markov chain Monte Carlo settings
☒	☐	For hierarchical and complex designs, identification of the appropriate level for tests and full reporting of outcomes
☒	☐	Estimates of effect sizes (e.g. Cohen's *d*, Pearson's *r*), indicating how they were calculated

Our web collection on statistics for biologists contains articles on many of the points above.

Software and code

Policy information about availability of computer code

Data collection	No software was used for data collection.
Data analysis	Programs for analyzing the MD simulations are located on Zenodo doi: 10.5281/zenodo.8360034. For plotting and statistical analysis, GraphPad Prism (version 10.0.2) was used.

For manuscripts utilizing custom algorithms or software that are central to the research but not yet described in published literature, software must be made available to editors and reviewers. We strongly encourage code deposition in a community repository (e.g. GitHub). See the Nature Portfolio guidelines for submitting code & software for further information.

Data

Policy information about availability of data

All manuscripts must include a data availability statement. This statement should provide the following information, where applicable:

- Accession codes, unique identifiers, or web links for publicly available datasets
- A description of any restrictions on data availability
- For clinical datasets or third party data, please ensure that the statement adheres to our policy

DATA AVAILABILITY. The authors declare that the data supporting the findings of this study are available within the paper and its supplementary information files, including source files. As the molecular dynamics simulation trajectories are of considerable size and cannot be deposited in freely available repositories, the authors have made available input files, from which the data can be generated. These input files can be accessed at Zenodo (doi: 10.5281/zenodo.8358517). Programs for analyzing the simulations can be found in a separate Zenodo archive (doi: 10.5281/zenodo.8360034).

CODE AVAILABILITY. The code used for the analysis of lipid scrambling and membrane disruption is available in public github repositories (links are provided in the Methods section under 'CGMD – Analysis').

Research involving human participants, their data, or biological material

Policy information about studies with human participants or human data. See also policy information about sex, gender (identity/presentation), and sexual orientation and race, ethnicity and racism.

Reporting on sex and gender	N/A
Reporting on race, ethnicity, or other socially relevant groupings	N/A
Population characteristics	N/A
Recruitment	N/A
Ethics oversight	N/A

Note that full information on the approval of the study protocol must also be provided in the manuscript.

Field-specific reporting

Please select the one below that is the best fit for your research. If you are not sure, read the appropriate sections before making your selection.

[x] Life sciences   [ ] Behavioural & social sciences   [ ] Ecological, evolutionary & environmental sciences

For a reference copy of the document with all sections, see nature.com/documents/nr-reporting-summary-flat.pdf

Life sciences study design

All studies must disclose on these points even when the disclosure is negative.

Sample size	Three independent replicas were considered as minimum for error estimation and this is commonly used for our methods. This is evident in graphs where individual data points are shown, and is also indicated in the figure legends. For MD simulations, the system size was chosen to avoid all known possible artefacts originating from the application of periodic boundary conditions.
Data exclusions	None.
Replication	The number of biological and technical replicates for each data set are indicated, as appropriate, in the figure legends.
Randomization	N/A. Randomization is not relevant to our study. Our samples/data were measured independently (an unbiased representation) and therefore did not need any further randomization.
Blinding	N/A. All the measurements are independent, leading to unbiased data points. Our study does not include any measurements/data from individuals.

Reporting for specific materials, systems and methods

We require information from authors about some types of materials, experimental systems and methods used in many studies. Here, indicate whether each material, system or method listed is relevant to your study. If you are not sure if a list item applies to your research, read the appropriate section before selecting a response.

Materials & experimental systems

n/a	Involved in the study	
[ ]	[x]	Antibodies
[ ]	[x]	Eukaryotic cell lines
[x]	[ ]	Palaeontology and archaeology
[x]	[ ]	Animals and other organisms
[x]	[ ]	Clinical data
[x]	[ ]	Dual use research of concern
[x]	[ ]	Plants

Methods

n/a	Involved in the study	
[x]	[ ]	ChIP-seq
[x]	[ ]	Flow cytometry
[x]	[ ]	MRI-based neuroimaging

Antibodies

Antibodies used	1. anti-6-His tag monoclonal antibody (MA1-21315, ThermoFisher)<br>2. anti-VDAC1/Porin Monoclonal antibody (ab110326, abcam)<br>3. anti-Mouse IgG, HRP-conjugate (W4021, Promega)<br>4. anti-rabbit IgG, HRP-conjugate (W4011, Promega)<br>5. anti-Psd1β 4077 (10.1016/j.isci.2021.102196; gift from S. Claypool)<br>6. anti-Tom70 (10.1002/j.1460-2075.1983.tb01553.x; gift from S. Claypool)
Validation	Commercial antibodies are validated by the supplier. Antibodies gifted by S. Claypool are validated in cited references.

Eukaryotic cell lines

Policy information about cell lines and Sex and Gender in Research

Cell line source(s)	Yeast strain TKY705 was a gift from Non Miyata (Kyushu University); mutant strains are from the same source or generated in this study. This information is provided in Table 7 of the Supplemntray Information.
Authentication	Drug selection (testing for the presence of the relevant strain marker) and PCR verification.
Mycoplasma contamination	N/A
Commonly misidentified lines<br>(See ICLAC register)	N/A

Plants

Seed stocks	N/A
Novel plant genotypes	N/A
Authentication	N/A
